# Metalloprotease Meprinβ in Rat Kidney: Glomerular Localization and Differential Expression in Glomerulonephritis

**DOI:** 10.1371/journal.pone.0002278

**Published:** 2008-05-28

**Authors:** Beatrice Oneda, Nadège Lods, Daniel Lottaz, Christoph Becker-Pauly, Walter Stöcker, Jeffrey Pippin, Maya Huguenin, Daniel Ambort, Hans-Peter Marti, Erwin E. Sterchi

**Affiliations:** 1 Institute of Biochemistry and Molecular Medicine, University of Bern, Bern, Switzerland; 2 Institute of Zoology, Johannes Gutenberg University, Mainz, Germany; 3 Division of Nephrology, University of Washington, Seattle, Washington, United States of America; 4 Division of Nephrology/Hypertension, Inselspital, University of Bern, Bern, Switzerland; Instituto Oswaldo Cruz and FIOCRUZ, Brazil

## Abstract

Meprin (EC 3.4.24.18) is an oligomeric metalloendopeptidase found in microvillar membranes of kidney proximal tubular epithelial cells. Here, we present the first report on the expression of meprinβ in rat glomerular epithelial cells and suggest a potential involvement in experimental glomerular disease. We detected meprinβ in glomeruli of immunostained rat kidney sections on the protein level and by quantitative RT-PCR of laser-capture microdissected glomeruli on the mRNA level. Using immuno-gold staining we identified the membrane of podocyte foot processes as the main site of meprinβ expression. The glomerular meprinβ expression pattern was altered in anti-Thy 1.1 and passive Heymann nephritis (PHN). In addition, the meprinβ staining pattern in the latter was reminiscent of immunostaining with the sheep anti-Fx1A antiserum, commonly used in PHN induction. Using Western blot and immunoprecipitation assays we demonstrated that meprinβ is recognized by Fx1A antiserum and may therefore represent an auto-antigen in PHN. In anti-Thy 1.1 glomerulonephritis we observed a striking redistribution of meprinβ in tubular epithelial cells from the apical to the basolateral side and the cytosol. This might point to an involvement of meprinβ in this form of glomerulonephritis.

## Introduction

Many forms of inflammatory kidney disease are characterized by excessive proliferation of mesangial cells (MC) and by increased deposition of extracellular matrix (ECM) in glomeruli and the tubulo-interstitium, with subsequent structural alteration of the glomerular basement membrane (GBM) and the mesangial matrix [1], [2]. Therefore, an analysis of the factors responsible for the regulation of MC proliferation and for the metabolism of ECM is of highest priority. The processing of ECM proteins in the kidney is influenced substantially by members of the metzincin superfamily of metalloendopeptidases, notably by the matrix metalloproteases (MMPs) [3]–[5] and by the ADAMs (a disintegrin and metalloprotease) [6], [7]. The role of MMPs in basement membrane remodelling in particular has recently received a lot of attention due to their ability to influence the progression of renal dysfunction [8]. Moreover, the pharmacological ablation of MMP enzymatic activity in a mouse model of Alport syndrome prior to the onset of proteinuria or structural defects of the GBM led to significant attenuation in disease progression and to prolonged survival [9]. Recently, the metalloendopeptidase meprin has been suggested to be involved in these processes, as well [10], [11]. Meprin (EC 3.4.24.18), an oligomeric zinc endopeptidase of the astacin family, was originally identified on the apical brush-border membrane of epithelial cells in the cortico-medullary portion of proximal kidney tubules and the intestine [12]–[15]. It is composed of α and β subunits that are expressed separately or coordinately either forming homo- or hetero-oligomeric complexes [16], [17]. Meprins are highly conserved among different species and are capable of cleaving a wide range of protein substrates *in vitro,* such as ECM components (collagen type IV, laminin, fibronectin and nidogen [18]–[21]), cytokines (interleukin-1 β), protein kinases, growth factors and peptide hormones [22]–[27]. Meprins act extracellularly in that they are either bound to the plasma membrane by transmembrane domains or secreted into the extracellular space [28], [29].

The role of meprin in the pathogenesis of kidney disease has been studied previously in animal models. There are mouse strains (ICR or C57B1/6) that express both meprinα and β in proximal tubular cells and others (C3H/He or CBA) that exclusively express meprinβ, mostly in a latent form (pro-meprinβ) with low proteolytic potential [30]. When exposed to different renal insults, the latter mice developed less severe forms of kidney disease compared to those with normal meprin levels, suggesting a potential role for meprin in the pathology of acute renal failure [31]. Moreover, in a model of ischemia and reperfusion injury, meprin was reported to be redistributed from the apical brush-border membrane to the basolateral side and the cytosol of tubular epithelial cells resulting in degradation of ECM and damage to adjacent peritubular structures [21]. Recently, it could be shown that this relocalization occurs also in a mouse model of acute kidney injury using cisplatin, suggesting that common mechanisms for meprin redistribution are involved in toxic and ischemic renal injury [32]. Furthermore, meprin was found to be directly cytotoxic to cultured tubular epithelial cells and its inhibition by actinonin and by the metal chelator 1,10-phenanthroline resulted in a protective effect on cell integrity during ischemia-reperfusion injury in rats [10], [33].

Multiple studies have also revealed that meprin expression is down-regulated in models of renal injury leading to acute renal failure. For example, renal damage associated with hydronephrosis (unilateral urethral obstruction) occurred with an early and progressive decline in rat meprin subunits on both mRNA and protein levels [34]. In addition, microarray analysis demonstrated marked declines in mouse meprinβ expression in acute renal ischemic reperfusion injury and in adriamycin-induced nephropathy [33], [35].

In a previous study using a rat model of chronic renal allograft rejection, we observed a concomitant down-regulation of meprinβ and MMP-9 [36]. MMP-9 has been shown to be secreted by both mesangial cells and glomerular epithelial cells (podocytes) [37]–[39]. We therefore decided to check for meprinβ expression in glomeruli and then to further assess meprin regulation in models of glomerular disease. For this purpose, we focused on meprinβ expression in healthy rat kidney and in two models of rat glomerular disease: passive Heymann nephritis (PHN), a rat model of human membranous nephropathy, and anti-Thy 1.1 mesangial proliferative nephritis [3], [40]–[42]. We demonstrate that a relocalization of the protease occurs following the induction of glomerular disease, an effect that may contribute to the pathogenesis of rat glomerulonephritis. Moreover, we demonstrate that meprin may represents a potential auto-antigen in PHN.

## Results

### Meprinβ expression in podocytes of rat glomeruli

**Figure 1:**
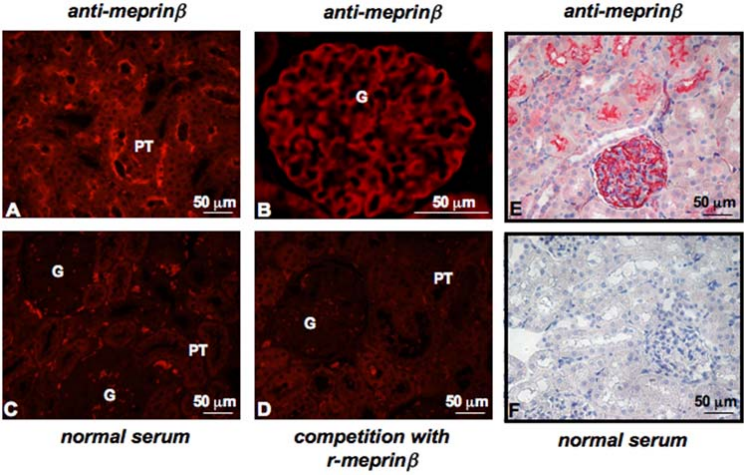
Meprinβ expression in Wistar rat kidneys. Immunostaining of paraffin embedded kidney tissues from Wistar rats: (A) positive immunofluorescence staining of proximal tubules and (B) of glomeruli using a specific meprinβ antibody. Control immunofluorescence staining: (C) replacement of the primary antibody with normal rabbit serum. (D) Competitive inhibition of meprinβ by pre-incubation with an excess of recombinant meprinβ (r-meprinβ). (E) Positive immunohistochemical staining of glomeruli using a specific C-terminal meprinβ antibody. Control immunohistochemical staining: (F) replacement of the primary antibody with normal rabbit serum. G: glomeruli, PT: proximal tubuli. Similar results were obtained using a different antibody directed against the N-terminal domain of meprinβ.

To assess meprinβ expression in healthy kidney paraffin-embedded kidney sections from Wistar rats were analyzed by immunostaining (Fig. 1). As expected, we observed strong apical staining of proximal tubular cells (PT) using a specific anti-meprinβ antibody (Fig. 1A, 1E). In addition, glomeruli (G) also displayed positive meprinβ staining (Fig. 1B, 1E). No signal was observed when the primary meprinβ antibody was replaced by normal rabbit serum (Fig. 1C, 1F), or following pre-incubation with a five-fold excess of recombinant human meprinβ (Fig. 1D). No effect on signal intensity was seen when the antibody was pre-incubated with bovine serum albumin (data not shown). We confirmed these data using Fisher and Lewis rats (supplementary Figure S1).

**Figure 2:**
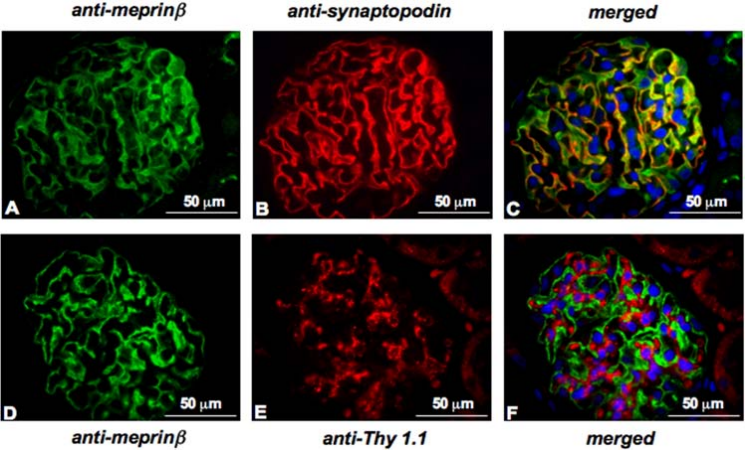
Double immunofluorescence staining of kidney sections from Wistar rats. Immunofluorescence staining of glomeruli: (A) anti-meprinβ antibody, (B) anti-synaptopodin antibody, (C) merged image meprin/synaptopodin, (D) anti-meprinβ antibody, (E) anti-Thy 1.1 antibody, (F) merged image meprin/Thy 1.1. Nuclei are marked by staining with 4,6-diamidino-2-phenylindole (DAPI).

To further investigate the localization of meprinβ in glomeruli, we double-stained the Wistar kidney sections with anti-meprinβ antibodies and anti-synaptopodin or anti-Thy 1.1 antibodies (Fig. 2). Synaptopodin is abundantly expressed in the foot processes of podocytes whereas Thy 1.1 is localized on the surface of glomerular mesangial cells. The staining pattern of meprinβ revealed co-localization with synaptopodin (Fig. 2A–C), but not with Thy 1.1 (Fig. 2D–F). Both meprinβ and synaptopodin displayed a linear staining pattern drawing the contour of the intra-glomerular capillaries. These findings clearly demonstrate the expression of meprinβ protein in podocytes of rat glomeruli.

### mRNA expression of meprinβ in glomeruli

**Figure 3:**
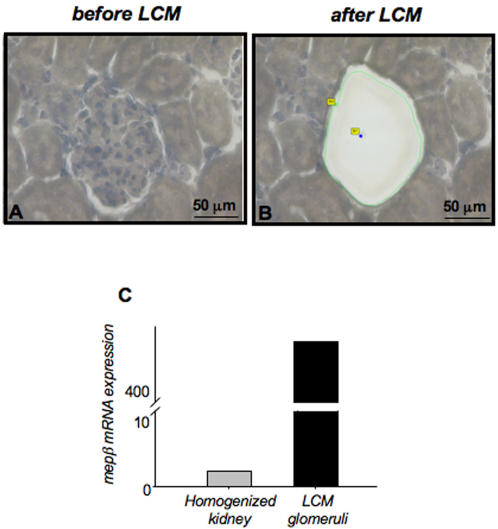
Relative meprinβ mRNA levels of laser-capture microdissected (LCM) glomeruli from Wistar rats. Two hundred glomeruli were selected for microdissection with a laser. Kidney cortex (A) before and (B) following LCM treatment. The tissue surrounding the captured glomeruli remains intact after microdissection. The presence of meprinβ was confirmed in the microdissected glomeruli by real-time quantitative PCR (C), and compared with the expression in the total homogenized kidney. mRNA levels were normalized with 18S as housekeeping gene.

Next, we analyzed the mRNA expression by real-time quantitative RT-PCR (RQ-PCR) on laser-capture microdissected glomeruli from Wistar rats (Fig. 3). Importantly, the tissue surrounding the captured glomeruli remained intact following microdissection (Fig. 3A, 3B). RQ-PCR analyses using 18S RNA as a house-keeping gene confirmed high meprinβ mRNA expression in glomeruli. The signal of meprinβ mRNA derived from the 200 collected glomeruli was 467-fold higher when compared to the level of the respective total kidney homogenate (arbitrarily set as 1.0-fold) (Fig. 3C), explained by the fact that all glomeruli contribute to meprin expression, while tubular meprinβ is confined to some proximal tubules of the cortico-medullary section and is therefore diluted in a whole kidney homogenate. The meprinβ mRNA results support the immunostaining data described above.

### Meprinβ in experimental PHN

**Figure 4:**
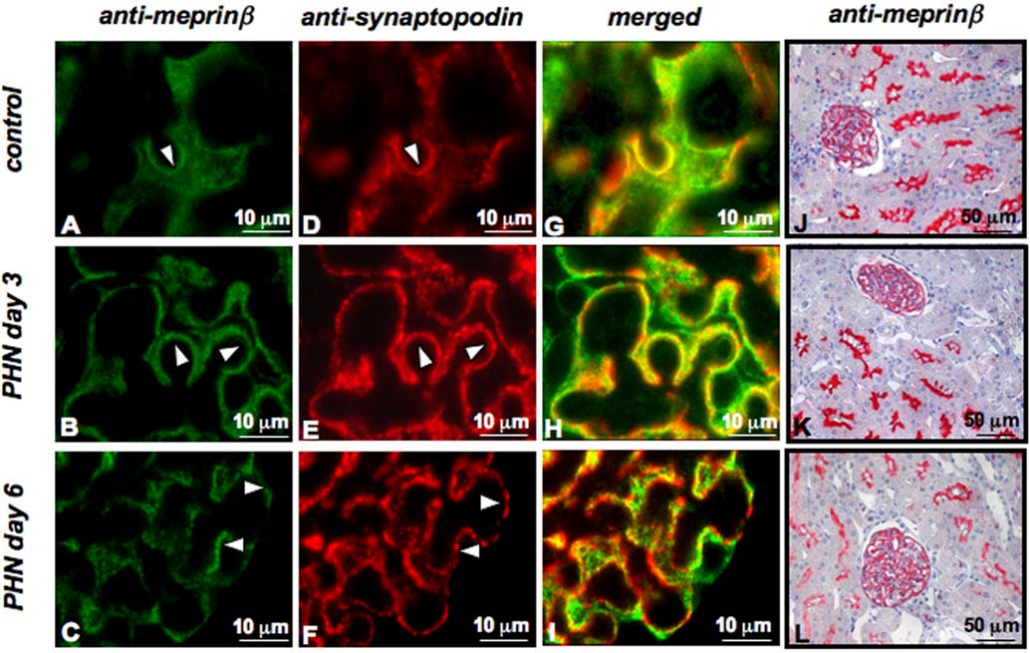
Meprinβ expression in rat glomeruli after induction of Passive Heyman nephritis (PHN). Immunofluorescence staining (A–C) of meprinβ and (D–F) synaptopodin in kidney sections from Sprague-Dawley rats. (G–I) Merged images of meprin/synaptopodin. (J–L) Immunohistochemical staining of meprinβ in glomeruli and in proximal tubules. Staining (A, D, G and J) in control rats, (B, E, H and K) in rats at day 3 and (C, F, I and L) in rats at day 6 after induction of PHN. A change in distribution of the meprinβ in PHN kidneys from a linear to a granular appearance was observed concomitantly associated with an overall reduction in signal intensity. The distribution of meprinβ and synaptopodin in glomeruli appeared increasingly divergent following progression of PHN.

PHN is a rat model of human membranous nephropathy associated with altered podocyte slit diaphragm integrity, sub-epithelial immune deposits and expansion of glomerular basal membranes [43], [44]. Analysis of kidney sections from control rats after injection of normal sheep globulin showed a linear staining pattern for meprinβ. This changed to a granular staining following injection of anti-Fx1A antiserum to induce PHN (Fig. 4A–C). At day 3, glomerular capillaries displayed a significant and well outlined meprinβ and synaptopodin staining, whereas at day 6, the staining intensity became granular and clustered and the overall picture was less intense in signal strength (Fig. 4B–F and Fig. 4 compare J and K to L), with the granular pattern being more pronounced for synaptopodin (Fig. 4G, 4H), probably as a result of a deterioration of podocyte integrity (Fig. 4I).

### Detection of meprinβ by anti-Fx1a antisera

**Figure 5:**
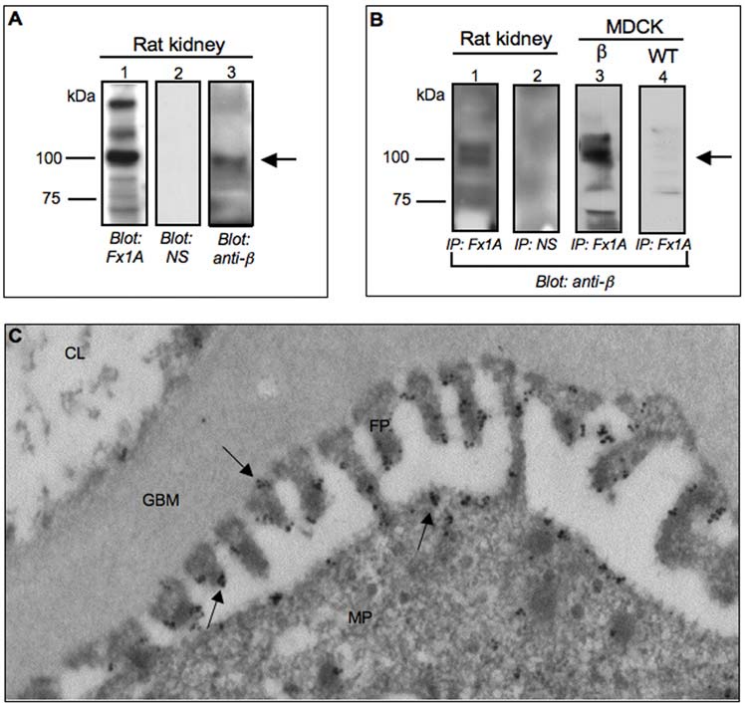
Meprinβ is recognized by anti-Fx1A antiserum. (A) Homogenized kidneys from Wistar rats were prepared and analyzed by Western blotting using sheep anti-Fx1A antiserum (lane 1), normal serum (NS) (lane 2) and meprinβ specific antibody (lane 3). A 110-kDa band was detected using the anti-Fx1A antiserum (lane 1) which corresponds to the meprinβ specific band (lane 3). No specific signal was detectable when normal serum was used as negative control (lane 2). (B) Anti-Fx1A antiserum was used to immunoprecipitate the proteins from homogenized rat kidneys or from the cell lysates of wild-type and human meprinβ-transfected MDCK cells. Normal sheep serum was used instead of anti-Fx1A antiserum as negative control. Immunoprecipitates were analyzed by Western blots using a specific meprinβ antibody. A 110-kDa fragment corresponding to meprinβ was found exclusively in the rat kidney (lane 1) and meprinβ-transfected MDCK cell lysates (lane 3) when immunoprecipitation was performed with anti-Fx1A antisera whereas no band was visualized with normal serum (lane 2). (C) Immuno-gold labeling shows meprinβ localization in podocyte major processes (MP) and foot processes (FP), as indicated by arrows. Meprinβ is located in the membrane of the podocyte. GBM: glomerular basal membrane. CL: capillary lumen.

Since the introduction of PHN as a model for auto-immune glomerulonephritis [45], a substantial effort was made to identify responsible antigens. It has been recognized that biologically relevant antigens must be present in the brush borders of the proximal kidney tubules as well as in podocyte membranes. PHN is induced by the administration of sheep or rabbit anti-Fx1A antisera generated against the brush border membrane fractions of rat proximal kidney tubules. Meprinβ is abundantly expressed in these membranes but, as shown above, also in glomeruli, the main target for the antibodies. Moreover, the staining pattern we observed for meprinβ resembled the one obtained by immunostaining using anti-Fx1A [46]. Hence, we checked if meprinβ was detected by the anti-Fx1A antiserum using Western blot analysis. In kidney homogenate from Wistar rats we detected a band of 110-kDa corresponding to the molecular size of meprinβ (Fig. 5A, lane 1). The same band was observed when the blot was stripped and re-probed with a specific anti-meprinβ antibody to show the specificity of the detection by anti-Fx1A (Fig. 5A, lane 3). In contrast, no band was detected when the membrane was blotted with normal serum as a negative control (Fig. 5A, lane 2). A 110-kDa band corresponding to meprinβ was also obtained after immunoprecipitation using anti-Fx1A antiserum, followed by Western blot analysis with a specific anti-meprinβ antibody (Fig. 5B, lane 1). No band appeared when the kidney homogenate was immunoprecipitated with normal serum as a negative control (Fig. 5B, lane 2). In addition, we stably transfected Madin-Darby canine kidney (MDCK) cells with human meprinβ. Wild-type and meprinβ transfected MDCK cells were then subjected to immunoprecipitation using anti-Fx1A antiserum, followed by Western blot analysis with anti-meprinβ antibody. We obtained a 110-kDa band in lysates of meprinβ-transfected MDCK cells whereas in wild-type MDCK cells this band was absent (Fig. 5B, line 3 and 5B, line 4). These findings clearly demonstrate that the anti-Fx1A antiserum, in addition to other brush border membrane proteins, detects meprinβ. Meprinβ could therefore be recognized at the podocyte membrane by the anti-Fx1A antiserum and thus contribute to the pathology of PHN as another auto-antigen.

To further strengthen this hypothesis, we analyzed the subcellular localization of meprinβ by immuno-gold labelling of ultra-thin sections of kidney cortex from adult Wistar rats using the specific anti-meprinβ antibody (Fig. 5C). Gold particles were found mostly at the membrane of podocyte foot processes and main processes, facing the urinary space and, to less extent, at the slit diaphragm. Meprinβ, an ectoenzyme, is thus accessible to circulating antibodies in rat glomeruli and this could lead to the formation and deposition of immuno-complexes and to the development of glomerulonephritis.

### Meprinβ in experimental anti-Thy 1.1 nephritis

**Figure 6:**
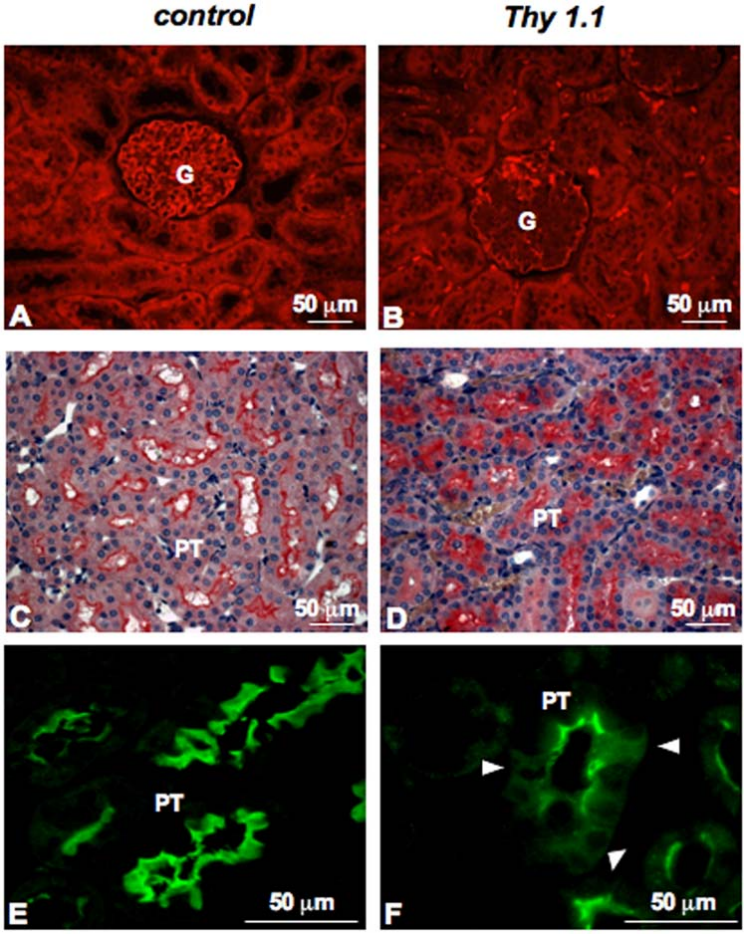
Meprinβ expression in experimental anti-Thy 1.1 nephritis. Meprinβ was detected by immunofluorescence and by immunohistochemistry in kidney sections (A, C and E) of healthy Wistar rats and (B, D and F) of Wistar rats at day 11 after induction of anti-Thy 1.1 nephritis. In nephritic rats, meprinβ expression is decreased in the glomeruli. Moreover, meprinβ expression is not only restricted to the brush border membrane of the tubules, but is also found diffusely spread within the tubular cells, as indicated by the arrow.

Anti-Thy 1.1 nephritis is characterized by an initial phase of complement-dependent mesangiolysis lasting for approximately 2 days, followed by a marked proliferative response of the residual mesangial cells associated with the accumulation of ECM [47]. We observed in preliminary experiments that the latter two features were reached at day 11 [48]. Therefore, this time point was chosen for nephrectomy and subsequent meprinβ immunostaining (Fig. 6). We observed a dramatic decrease in meprinβ expression in all glomeruli tested at day 11 after anti-Thy 1.1 injection (Fig. 6B). In contrast, in control rats injected with PBS we observed normal meprinβ protein levels (Fig. 6A). This observation is in accordance with the alterations in glomerular structure featuring various glomerular diseases, particularly at the level of podocytes. Substantial damage to glomeruli was also reflected by a distorted synaptopodin staining, indicating loss of podocyte integrity (data not shown). These changes in protein levels occur along with the onset of proteinuria, having a maximum around day 2 and 3 after induction of disease. Moreover, we also noticed a different staining pattern in proximal tubules: in control rats, a strong signal was localized at the apical plasma membrane, whereas kidney sections of nephritic rats displayed a more diffuse staining throughout the tubular epithelial cells (Fig. 6D–6F). These findings point to a differential and rapid relocalization of meprinβ towards the basolateral side and the cytoplasm of proximal tubular cells during mesangioproliferative glomerulonephritis.

## Discussion

Meprin comprises approximately 5% of the brush border membrane in rodent kidney, only second in abundance to actin [49]. Hence it is considered to be the major protein of the microvillar membrane with activity for degrading extracellular matrix proteins [21]. Numerous studies have demonstrated that renal meprin expression plays a key role in the severity and progression of renal injury, with meprin exacerbating the disease.

In our study we detected for the first time meprinβ expression in the glomeruli of three different rat strains, a finding expanding our knowledge on meprin's previously described expression in the apical membrane of proximal tubular epithelial cells. Interestingly, a metalloendopeptidase with a molecular weight corresponding to that of meprin was previously found in rat glomeruli, but was not identified [50]. Using specific markers for glomerular cells and immuno-gold staining we identified the membrane of podocyte foot processes as the main site of glomerular expression of meprinβ.

In two different rat models of glomerulonephritis we detected changes in the expression pattern of meprinβ, both in the glomeruli as well as in the proximal tubules. In passive Heymann nephritis (PHN), the immunofluorescence signal for meprinβ in glomeruli paralleled that of synaptopodin. An increasingly granular staining was observed indicative of the disruption in structural integrity of the podocyte foot processes. In addition, the intensity of meprinβ staining was decreased both in the glomeruli and in the proximal tubules with disease progression. PHN is induced by the administration of antibodies generated against a membrane fraction of rat renal proximal tubular cells (sheep or rabbit anti-Fx1A antibodies), which results in the formation and deposition of sub-epithelial immune complexes at the glomerular basement membrane (GBM) [51]. This leads to the disruption of the functional integrity of the GBM and the filtration barrier, which in turn results in proteinuria. Megalin, a multiligand endocytic receptor, localized in the microvillus membrane of proximal tubular epithelial cells, was previously identified as an antigen in these immune deposits [52]. The observation of identical staining patterns with anti-meprinβ and anti-rat Fx1A antibodies [46], the direct detection of meprinβ by anti-Fx1A antibody in Western blots and in immunoprecipitation assays, and the subcellular localization of meprinβ in the membrane of podocyte foot processes all suggest that meprinβ may be involved in the pathogenesis of PHN by representing another auto-antigen. The decrease of meprinβ immunostaining signal in glomeruli from rats with PHN may reflect the typical injury of podocytes in this disease or may reflect a reduced binding affinity of the staining antibody to the meprinβ-immunocomplexes.

Physiologically, the integrity and the structure of the glomerular basement membrane (GBM) is maintained by the podocytes in a continuous replacement and recycling effected by proteases [53]–[56]. Meprin, expressed by podocytes, may act as such a processing entity in breaking down GBM components, prior to being substituted by new matrix proteins. A plethora of ECM components are processed by meprin *in vitro*, such as laminin, collagen IV, nidogen and fibronectin [19]–[21]. Damage to the podocytes, either directly in PHN or indirectly through mesangial injury in Thy 1.1 nephritis, may then interfere with the normal turnover function of meprin and may thus disturb the steady-state processing of GBM components and hence enhance progression of the disease.

Thy 1.1 nephritis is a reversible glomerular disease that affects mesangial cells. We observed a loss of meprinβ signal in rat podocytes at day 11 following induction of the disease. We postulate that the damage occurring in the mesangial compartment may also lead to changes in podocytes in Thy 1.1 nephritis. In addition, we observed a marked alteration in meprinβ staining of proximal tubular cells despite the fact that Thy 1.1 nephritis is a glomerular disease: the immunostaining revealed a shift from a linear staining confined to the apical brush border membrane in the corticomedullary junction towards a diffuse cytoplasmatic staining involving the tubular basement membrane. These findings are reminiscent of meprinβ relocalizations reported in tubular nephritis such as in hypoxia/reperfusion injury [10], [21], [31] or in an acute kidney injury model of cisplatin nephrotoxicity [32]. These data provide evidence that high levels of meprin are present in locations other than the apical membrane exposing the protease to a distinct set of substrates not normally available to meprinβ. These include proteins of the basement membranes and cell-cell adhesion complexes. It has been shown that mice with low renal meprin expression levels had less severe forms of structural injury when compared to mice with higher meprin levels [31], [57]. Therefore, expression of meprin in epithelial cells of proximal tubules appears to be detrimental in an already stressed kidney and thus worsens the disease course.

This report is to our best knowledge the first to show expression of the metalloendopeptidase meprinβ in rat glomeruli. Moreover, our results suggest that localization, balance and regulation of meprinβ may constitute features in kidney disease progression. In order to investigate whether meprinβ also has a direct, causal role in the pathogenesis of glomerulonephritis such as PHN, further experiments using animal models are needed. Moreover, it will be interesting to correlate our results with studies in human glomerulonephritis to assess a role of meprinβ as a possible auto-antigen in membranous nephropathy.

## Materials and Methods

### Animal tissues

Paraffin-embedded kidney tissues from Fisher and Lewis rat strains were kindly provided by collaborators in Leiden, The Netherlands. Approval for the studies was obtained from the local animal studies commission.

The rats were sacrificed by intra-peritoneal injection of 1 ml of pentobarbital. After nephrectomy the kidneys were either snap frozen in liquid nitrogen and stored at -80°C, or fixed for 24 hours in 5% buffered formalin, de-hydrated and embedded in paraffin.

### Induction of disease

Anti-Thy 1.1 nephritis was induced at day 0 in Wistar rats (150 g body weight) by an intravenous injection of anti-Thy1.1 IgG (OX-7; 1 mg/kg body weight in 500 µl PBS) as previously described [48]. Kidneys were harvested 11 days after injection, fixed in formalin and embedded in paraffin.

PHN was induced in male Sprague-Dawley rats (weighing 180 g to 200 g) by intra-peritoneal injection (5 ml/kg body weight) of sheep anti-Fx1A antibody prepared as described previously [58], [59]. Rats were sacrificed 3 or 6 days after induction of nephritis.

### Immunohistology

Immunohistochemistry of meprinβ on rat kidney section was performed as described previously [36]. Replacement of the primary antibody with normal rabbit serum served as a control for the staining specificity (DAKO Diagnostics, Zug, Switzerland).

### Immunofluorescence

The paraffin-embedded kidney sections were de-waxed, re-hydrated and pre-treated by heating in a microwave oven in 10 mM citrate buffer, pH 5.65. Thereafter, sections were washed with PBS and incubated in 5% bovine serum albumin blocking solution, before further incubation with the primary antibody for 1 hour at room temperature. Sections were then incubated with fluorescence labelled secondary antibody for 45 min at room temperature in the dark, 4,6-diamidino-2-phenylindole (DAPI) stained for 2 min, mounted in Dako Cytomation Fluorescence-Mounting Medium and sealed with a cover slip. To prove specificity of the antisera, we either re-placed the primary antibodies with normal rabbit serum (DAKO Diagnostic) or we pre-incubated the primary antibody with recombinant meprinβ (competitive inhibition) [60]. For double labelling, we applied a second primary antibody for 1 hour. We used the following antibodies: primary rabbit polyclonal anti-meprinβ, targeting amino acids 468–612 of meprinβ, as previously described [61], primary mouse monoclonal anti-synaptopodin (Progen, Heidelberg, Germany), primary monoclonal mouse anti-Thy 1.1 (Oxford Biotechology, UK), secondary goat anti-rabbit and goat anti-mouse (Dako Cytomation, Denmark), diluted 1:500, 1:4, 1:20, 1:1000 and 1:400 respectively.

### Immunoelectron microscopy

Isolated rat kidneys were fixed in 0.1% glutaraldehyde and 3% paraformaldehyde in 0.1 M phosphate buffer (pH 7.4) for 3 hours at room temperature. Fixed tissue was dehydrated to 96% ethanol, embedded in LR White hard (Science Services, Munich, Germany), and polymerized at 4°C under ultraviolet (UV) light for 48–60 hours. Ultrathin sections (50 nm) were cut on a Leica Ultracut S and were collected on Formvar-coated nickel grids. Sections were first etched with saturated sodium periodate (Sigma) at room temperature for 2 min. The grids were pre-incubated with 0.1% Tween 20 in phosphate-buffered saline (PBS), then blocked with 50 mM NH_4_Cl in PBS and in blocking solution (0.5% fish gelatine (Sigma) plus 0.1% ovalbumin (Sigma) in PBS). Sections were incubated with primary antibodies against meprinβ diluted in blocking solution at 4°C for 60 hours, washed in PBS and twice in a mixture of 0.1% ovalbumin, 0.5% cold-water fish gelatin, 0.01% Tween 20, 0.5 M NaCl in 10 mM phosphate buffer, pH 7.3 (IgG-gold buffer). The sections were incubated for 2 hours with goat anti-rabbit Fab conjugated to nanogold™ (1.4-nm gold particles; Nanoprobes, Stony Brook, NY), diluted in IgG-gold buffer. Washed sections were postfixed in 1% glutaraldehyde for 5 min and air-dried. The nanogold™ labeling was silver-enhanced for 25 min at room temperature as described [62]. The grids were then washed in distilled water and stained with 2% ethanolic uranyl acetate for 10 min and with lead citrate for 2 min before examination in a FEI Tecnai 12 TEM [63].

### Laser-capture microdissection (LCM) and reverse transcription

Kidneys of Wistar rats were embedded individually in polyethylene glycol freezing media (Tissue-Tek OCT Compound, Sakura Finetek, Torrance, CA, USA), placed at -20°C for 1 hour and then stored at −80°C. Tissues were sectioned and mounted on PALM PEN-covered membrane slides (PALM AG, Bernried, Germany). LCM was performed immediately after sectioning. Sections were stained with hematoxylin for 20 s using the HistoGene Staining Solution (Arcturus HistoGene, LCM Frozen Section Staining Kit, CA, USA) and dehydrated in graded solutions of ethanol for 30 s each. Once air-dried, 200 glomeruli were microdissected and catapulted into PALM adhesive caps tubes using the laser microdissector pressure catapulting device (PALM AG, Bernried, Germany). Cells were lysed in RNA extraction buffer and total RNA was extracted using the PicoPure RNA Isolation Kit (Arcturus). Total RNA was reverse-transcribed according to the manufacturer's instructions (Promega, Catalys AG, Wallisellen, Switzerland).

### Real-time quantitative RT-PCR (RQ-PCR)

cDNA was used as a template for RQ-PCR analysis with the Applied Biosystems 7500 PCR System according to the manufacturer's protocol in a reaction volume of 20 µl containing 1x TaqMan Universal PCR Master Mix and 1x TaqMan gene expression assay (Applied Biosystems Rotkreuz, Switzerland). All the target genes were measured in duplicates. To detect genomic DNA contamination, all analyses were also carried out without reverse transcriptase and without template as negative controls. 18S RNA expression levels served as a housekeeping gene to normalize expression between different samples and to monitor assay reproducibility. Relative quantification of all targets was calculated by the comparative cycle threshold method outlined by the manufacturer (User Bulletin No. 2; Applied Biosystems).

### Immunoblot and immunoprecipitation assay

Homogenized kidney from Wistar rats or cell pellets of the stably transfected MDCKβ and WT [64] were lysed in buffer A (25 mM Tris/HCl pH 8, 50 mM NaCl) containing 1% deoxycholic acid and 1% Nonidet P-40 in the presence of protease inhibitors for 30 min on ice. Protein extracts were then denatured by boiling 5 min with SDS 0.5% followed by electrophoresis on 7.5 or 10% SDS-polyacrylamide gels and transfer to polyvinylidene difluoride membranes (Hybond–P PVDF, Amersham Biosciences, Uppsala, Sweden). The membranes were saturated with 5% dry milk and 0.1% Tween-20 in Tris-buffered saline for 2 hours, incubated overnight with the Fx1A antibody (1:1000) or the specific meprinβ antibody (1:2000) and then incubated with horseradish peroxidase-conjugated anti-sheep IgG (1:5000) for 1.5 hours at room temperature. Immunocomplexes were visualized using the ECL Plus Western blotting kit (Amersham Biosciences) and X-ray films.

For immunoprecipitation, denatured protein extracts were incubated with 50 µl of protein G-Sepharose beads conjugated with the Fx1A antibody for 30 min at 4°C. Immune complexes were pelleted by microcentrifugation at 15000 g for 5 min at 4°C and washed 4 times with 0.5% Nonidet P-40, 0.05% deoxycholic acid and 0.05% SDS in NaCl/P_i_. The immunoprecipitate was then subjected to immunoblotting using anti-meprinβ antibodies as described above.

## Supporting Information

Figure S1 Meprinβ expression in Fisher and Lewis rat kidneys. Immunostaining of paraffin-embedded kidney sections (A and C) from Fisher and (B and D) Lewis rats. Positive staining of glomeruli and proximal tubules (A and B) in immunohistochemical sections and (C, D) in immunofluorescence sections using the C-terminal anti-meprinβ antibody.
(2.15 MB DOC)
