# Supplementary material for: Metalloprotease Meprinβ in Rat Kidney: Glomerular Localization and Differential Expression in Glomerulonephritis
Source: PLoS One. 2008 May 28;3(5):e2278. doi: 10.1371/journal.pone.0002278 (PMC2386549; doi:10.1371/journal.pone.0002278)
Supplement: Figure S1 — Meprinβ expression in Fisher and Lewis rat kidneys. Immunostaining of paraffin-embedded kidney sections (A and C) from Fisher and (B and D) Lewis rats. Positive staining of glomeruli and proximal tubules (A and B) in immunohistochemical sections and (C, D) in immunofluorescence sections using the C-terminal anti-meprinβ antibody. (2.15 MB DOC) [file pone.0002278.s001.doc]

**Supplementary figure 1**

**Meprinβ expression in Fisher and Lewis rat kidneys.** Immunostaining of paraffin-embedded kidney sections **(A and C)** from Fisher and **(B and D)** Lewis rats. Positive staining of glomeruli and proximal tubules **(A and B)** in immunohistochemical sections and **(C, D)** in immunofluorescence sections using the C-terminal anti-meprinβ antibody.


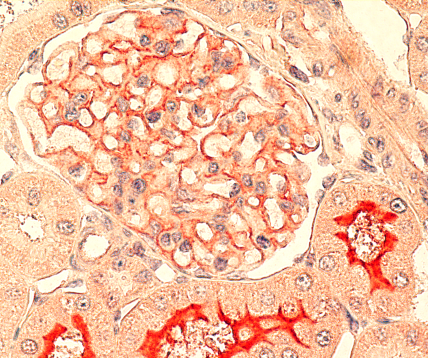

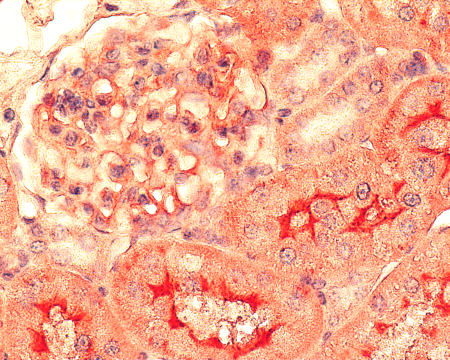

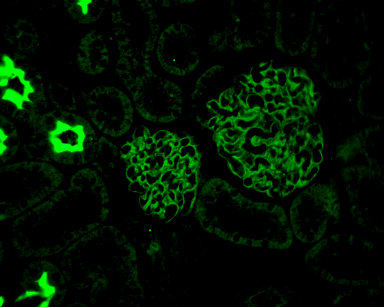

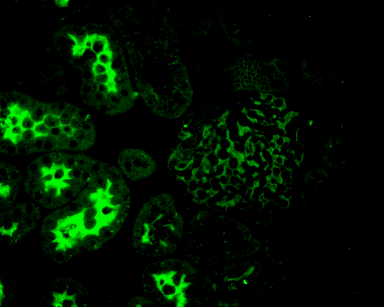


**PT**

**G**

G

**PT**

**G**

**G**

**PT**

**PT**

**G**

***Fisher rat***

***Lewis rat***

**50 μm**

**50 μm**

**50 μm**

**50 μm**

**C**

**D**

**B**

**A**
